# Supplementary material for: High throughput platform technology for rapid target identification in personalized phage therapy
Source: Nat Commun. 2024 Jul 11;15:5626. doi: 10.1038/s41467-024-49710-2 (PMC11239838; doi:10.1038/s41467-024-49710-2)
Supplement: Supplementary file 1 — Supplementary Information [file 41467_2024_49710_MOESM1_ESM.pdf]

# *Supplementary Information*

## High throughput platform technology for rapid target identification in personalized phage therapy

*Fereshteh Bayat,*[a] *Arwa Hilal,*[a] *Mathura Thirugnanasampanthar,*[b] *Denise Tremblay,*[c,d,e] *Carlos D. M. Filipe,*[b] *Sylvain Moineau,*[c,d,e]

*Tohid F Didar,*[a,f,g †] *Zeinab Hosseinidoust*[a,b,g,h†]

[a] School of Biomedical Engineering, McMaster University, Hamilton, Ontario, L9S 8L7, Canada

[b] Department of Chemical Engineering, McMaster University, Hamilton, Ontario, L9S 8L7, Canada

[c] Département de biochimie, de microbiologie et de bio-informatique, Faculté des sciences et de génie, Université Laval, Québec City, QC, G1V 0A6, Canada

[d] Groupe de recherche en écologie buccale, Faculté de médecine dentaire, Université Laval, Québec City, QC, G1V 0A6, Canada

[e] Félix d'Hérelle Reference Center for Bacterial Viruses, Université Laval, Québec City, QC, G1V 0A6, Canada

[f] Department of Mechanical Engineering, McMaster University, Hamilton, Ontario, L9S 8L7, Canada

[g] Michael DeGroote Institute for Infectious Disease Research, McMaster University, Hamilton, Ontario, L98 4L8, Canada

[h] Farncombe Family Digestive Health Research Institute, McMaster University, Hamilton, Ontario, L8S 4K1, Canada

[†]Corresponding Author. Phone: (905) 525-9140; email: didar@mcmaster.ca; doust@mcmaster.ca

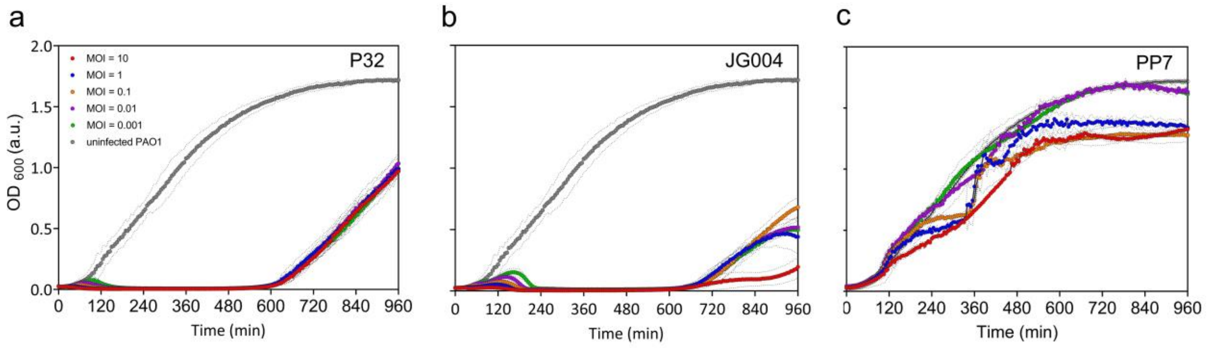


**Supplementary Figure 1. Optical density ($OD_{600}$) or growth curve of *Pseudomonas aeruginosa* PAO1 strain (Pa). a** Pa infected with P32 shows a strong performance in preventing bacterial growth by decreasing the culture turbidity even at lower MOIs (0.001). **b** Pa infected with JG004 showed effective bacterial growth suppression **c** Pa infected with PP7 showed week efficacy is preventing bacterial growth which aggravates by decreasing the initial concentration of phage (lower MOIs). The represented data are the average of three independent experiments (n=3) with at least 6 technical replicates. Source data are provided in Source Data file.

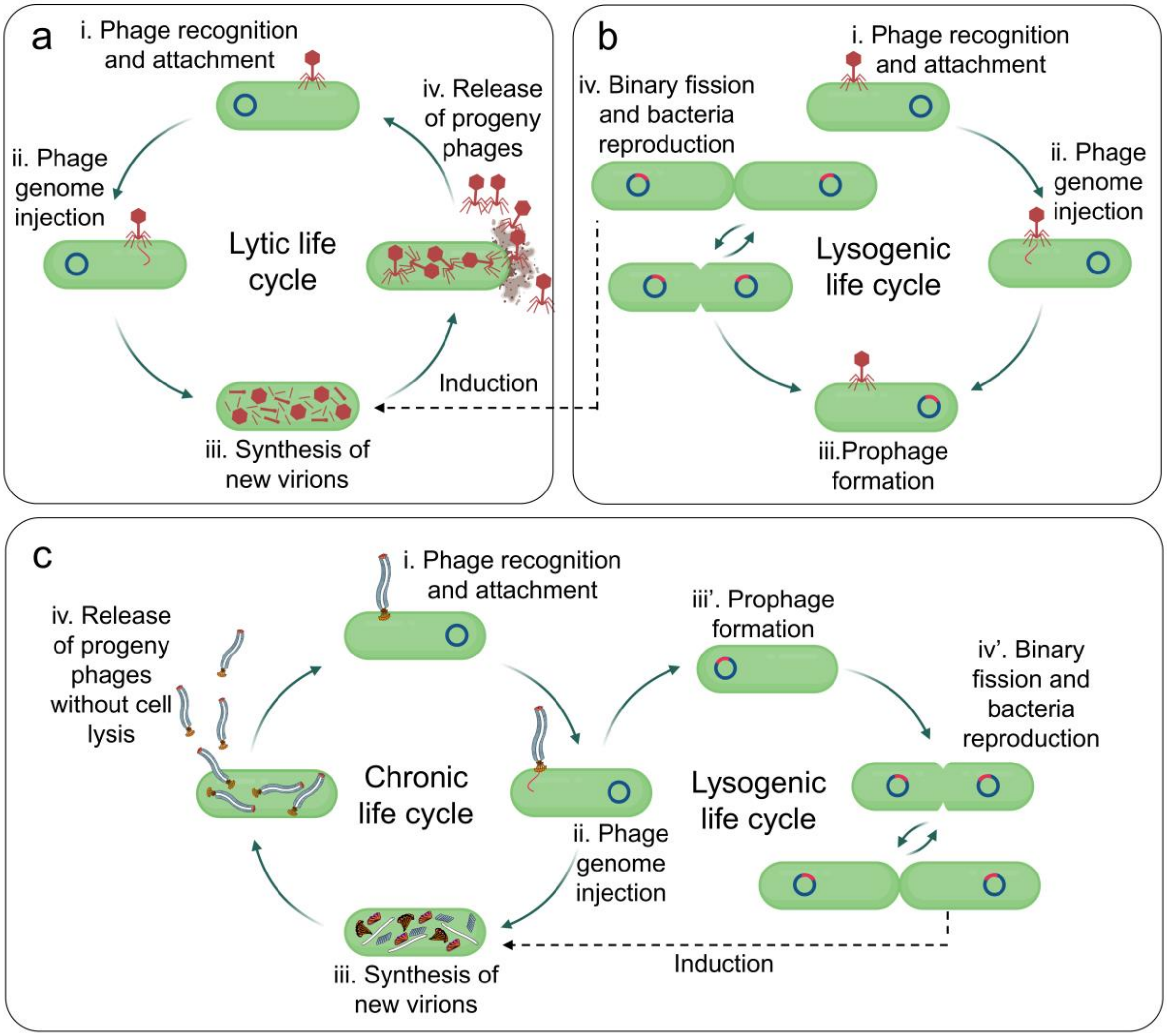


**Supplementary Figure 2. Schematics of phage life cycles. a** Lytic life cycle. The lytic cycle of phages starts with (i) phage recognition and attachment to bacterial cell receptors, (ii) genome injection, (iii) taking control of the bacterial replication machinery and producing new virions, and (iv) release of progeny phages by bacterial cell lysis. **b** Lysogenic life cycle. In the lysogenic life cycle, phage also adsorbs to cell receptors (i) and injects its genome inside the bacterial cell (ii); however, the phage genome is integrated into the bacterial genome, forming a prophage (iii) that is replicated through the bacterial reproduction cycle (iv). The lysogenic cycle could be induced to start the lytic cycle with environmental triggers such as high temperature or UV exposure which can ultimately lead to cell lysis (as shown by the dashed arrow). **c** Chronic life cycle. Similar to other phage life cycles, the chronic lifestyle (mostly seen in filamentous phages) starts with recognition and adsorption to bacterial cell receptors (i) and genome injection (ii). By taking control of the bacterial replication machinery, new virions are synthesized inside the bacterial cell (iii) and are released through budding or extrusion without lysing bacterial cells (iv). Some chronic phages can also adopt a lysogenic lifestyle, in which the phage genome is incorporated into the bacterial genome forming a prophage (iii′) and is replicated through the bacterial reproduction cycle and staying dormant (iv′) until the chronic cycle is induced by environmental stimuli (shown by the dashed arrow). Panels a and b Created with BioRender.com released under a Creative Commons Attribution-NonCommercial-NoDerivs 4.0 International license.

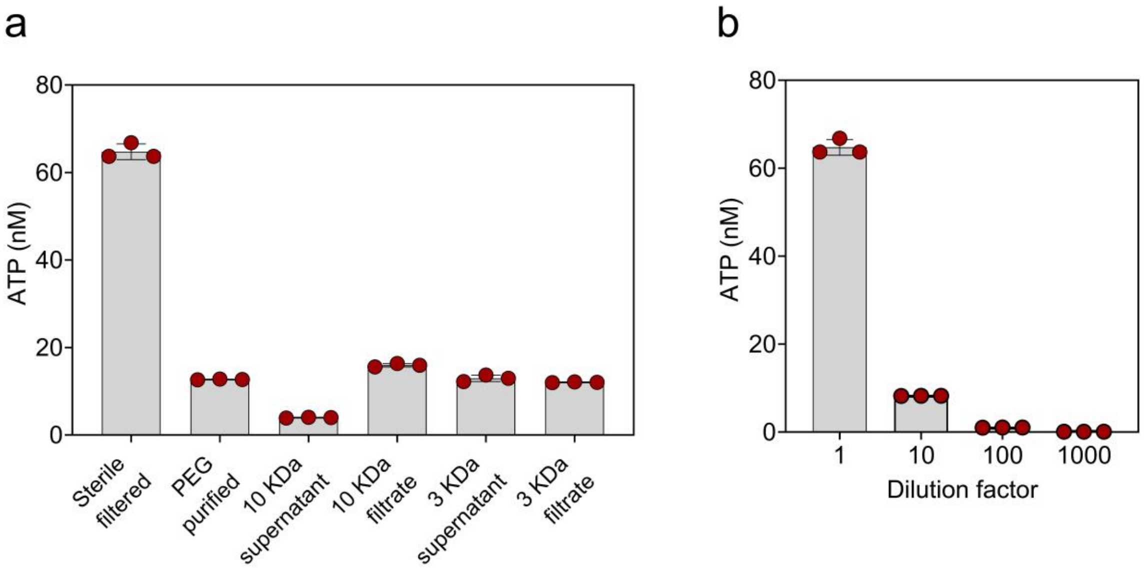


**Supplementary Figure 3. Decreasing ATP background in phage solutions. a** ATP background at different stages of P32 phage purification. PEG purification decreased the ATP background in phage solution. 10 KDa ultrafiltration decreased the ATP background significantly, while 3 KDa filters failed to reduce ATP levels. **b** Effect of dilution of phage suspension in bacterial cell culture media (LB media) on ATP background. The effect of diluting phage samples is comparable to purifying phage suspensions. Depending on the availability, either diluted filtered phages in bacterial cell media/buffer or purified phages can be used to reduce the effect of the background bioluminescence signal intensity. Results shown are the average of three replicates (n=3) with associated error bars showing standard deviation from the mean. Source data are provided in Source Data file.

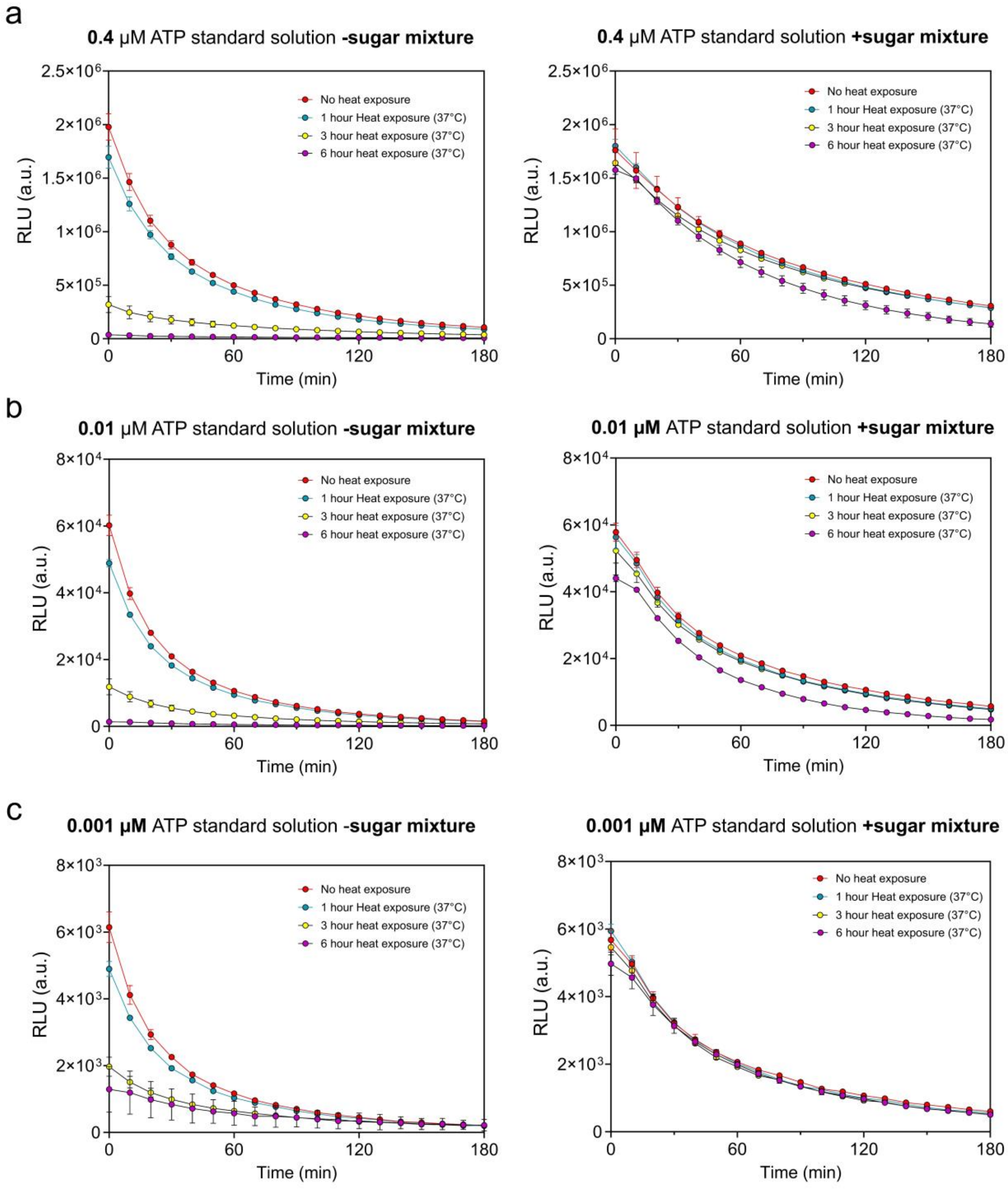


**Supplementary Figure 4.** Effect of sugar polymers on stability of the ATP reagent solution at 37°C. ATP standard solutions were added to ATP reagent solution with (+) and without (-) sugar mixture at time =0, after incubating for one, three and six hours at 37°C, and the results were compared to no heat exposure ATP reagent solution. ATP standard solutions are used at **a** 0.4 µM, **b** 0.01 µM, and **c** 0.001 µM. The RLU signal was measured kinetically every 10 minutes for up to 3 hours. Sugar mixture was able to preserve the activity of luciferin and luciferase as there were no significant changes in the RLU signals after heat exposures. In the absence of sugars, the

activity of ATP reagent solution decreased as significantly lower RLU signals were detected, which was more drastic after 3 and 6 hours of heat exposure. The RLU signal also dropped faster in the absence of sugar mixture. The presented data are the average of three replicates (n=3) with error bars representing standard deviation from the mean. Source data are provided in Source Data file.

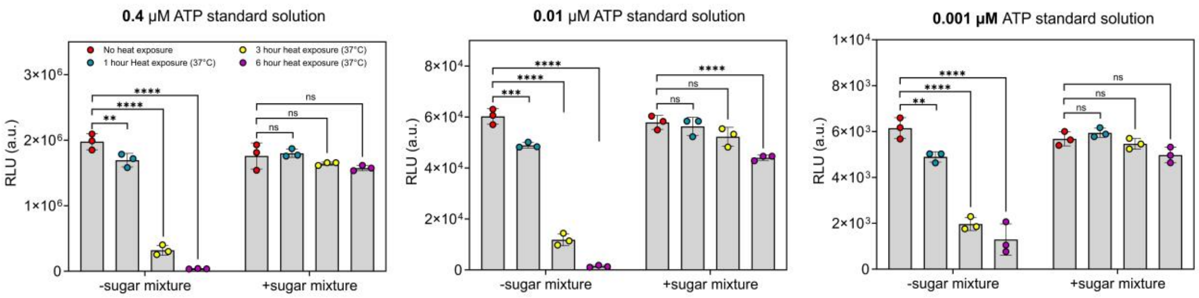


**Supplementary Figure 5. Comparison of the RLU signal intensity of heat treated and untreated ATP reaction solutions in the presence and absence of sugar mixture after heat exposure at 37°C.** RLU signal at time=0, right after adding ATP standard solution with 0.4 µM, 0.01 µM, 0.001 µM to ATP reagent solutions with (+) and without (-) sugar mixture after incubating for 1, 3 and 6 hours at 37°C in comparison with no heat exposure ATP reagent solution (time=0). The presented data are the average of three replicates (n=3) with Standard deviation from the mean. Statistical significance in all panels is derived from Two-way analysis of variance (ANOVA). Significance levels include *$P$ < 0.05, **$P$ < 0.01, ***$P$ < 0.001, and ****$P$ < 0.0001. Source data are provided in Source Data file.

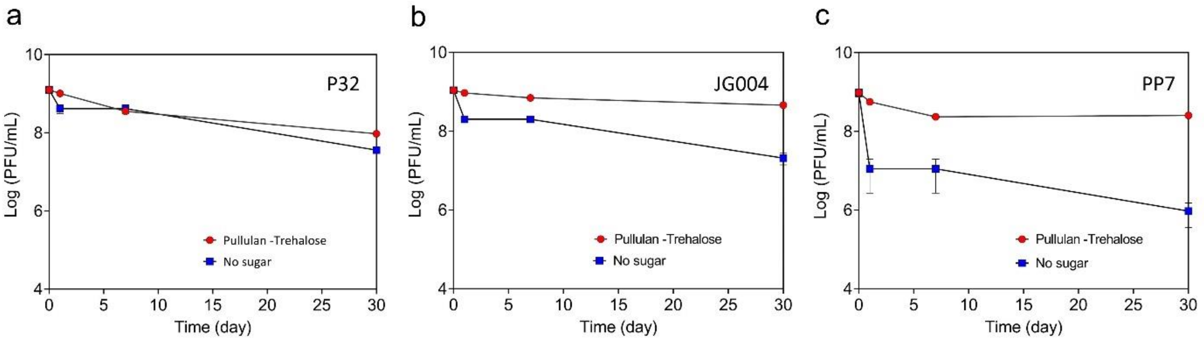


**Supplementary Figure 6.** Stability of dried *Pseudomonas* phages P32 (a), JG004 (b), and PP7 (c) in ambient conditions in 10 wt% pullulan + 0.5 M trehalose in comparison with no sugars. Sugar polymers helped to retain higher infectivity after 30 days. The presented data are the average of three replicates (n=3) with Standard deviation from the mean. Source data are provided in Source Data file.

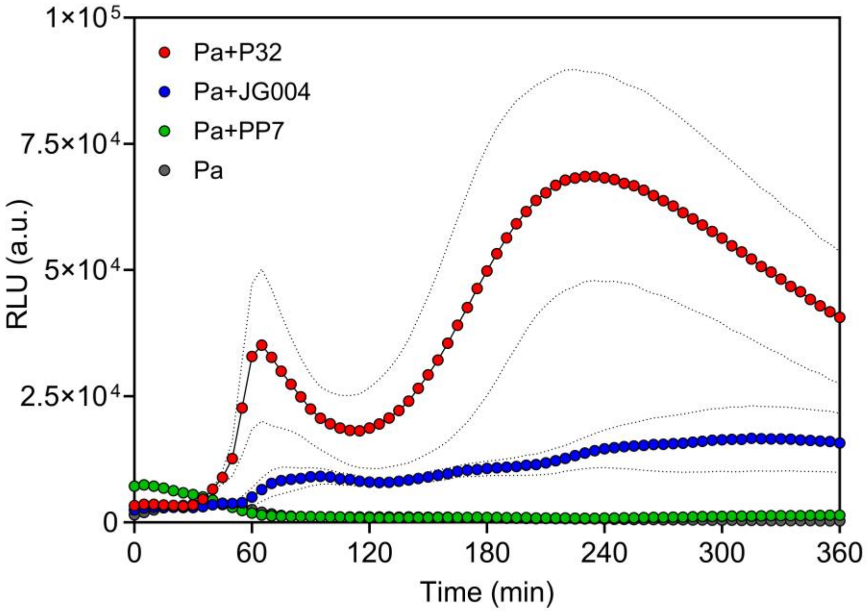


**Supplementary Figure 7. Phage and ATP reagents stability assay after 4 weeks.** The all-inclusive tablets including phages and ATP reagent solutions in sugar polymer matrices were stored for 4 weeks at room temperature under vacuum. To run the assay, the tablets were reconstituted with sterile Milli-Q water, followed by addition of *P. aeruginosa* (Pa) subcultures at $OD_{600}$ ~0.1 and kinetic monitoring of RLU signal. The data presented are the average of 6 replicates, and dashed line showing standard deviation from the mean. Source data are provided in Source Data file.

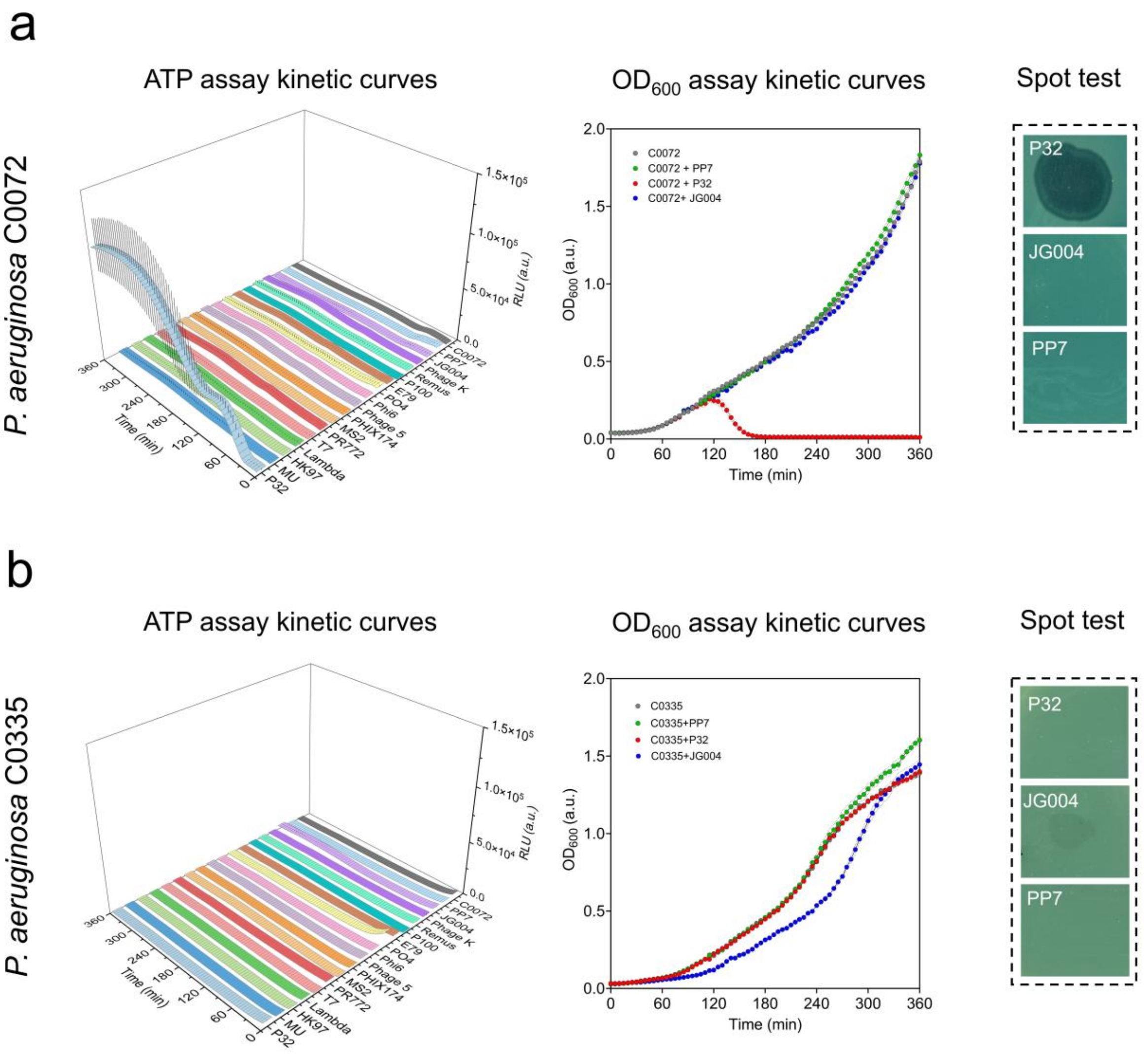


**Supplementary Figure 8. Screening Clinical *P. aeruginosa* isolates against an in-house phage library. a** C0072 strain isolated from a patient with urinary tract infection screened against a library of phages including phages from different species (**Supplementary Table 1**) using desiccated Sugar-based one-pot ATP bioluminescence assay, optical density assay ($OD_{600}$), and representative spot test on C0072 bacterial lawn. **b** C0335 strain isolated from a patient with arm infection, screened against an in-house phage library using one-pot ATP bioluminescence assay optical density assay ($OD_{600}$) and spot test on C0335 bacterial lawn. The $OD_{600}$ assay and spot test was conducted on three P. aeruginosa phages including P32, JG004, and PP7. ATP and OD assay were conducted in triplicates (n=3). The dashed lines show standard deviation from the mean in $OD_{600}$ assays and the error bars in ATP assays are standard deviation from the mean. Source data are provided in Source Data file.

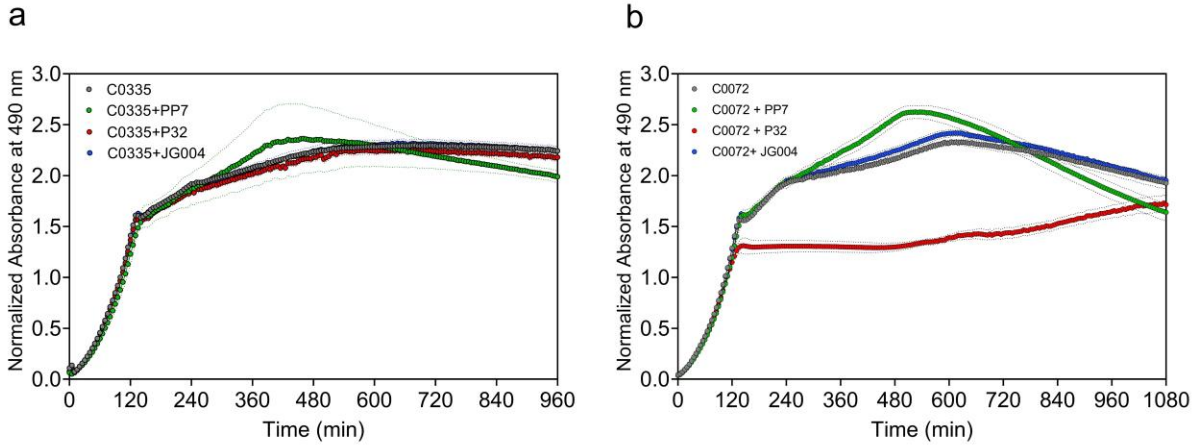


**Supplementary Figure 9. Metabolic activity measurement of clinical isolates of *P. aeruginosa* with P32, JG004, and PP7 at MOI ~ 10. a** Metabolic activity of phage infected C0335. There were no significant changes in metabolic activity of phage infected C0335 compared to uninfected C0335. **b** Metabolic activity of phage infected C0072. metabolic activity of C0072 decreased after 2 hours for C0072 infected with P32. The data presented are the average of at least 3 replicates (n=3). The dashed lines show standard deviation from the mean. Source data are provided in Source Data file.

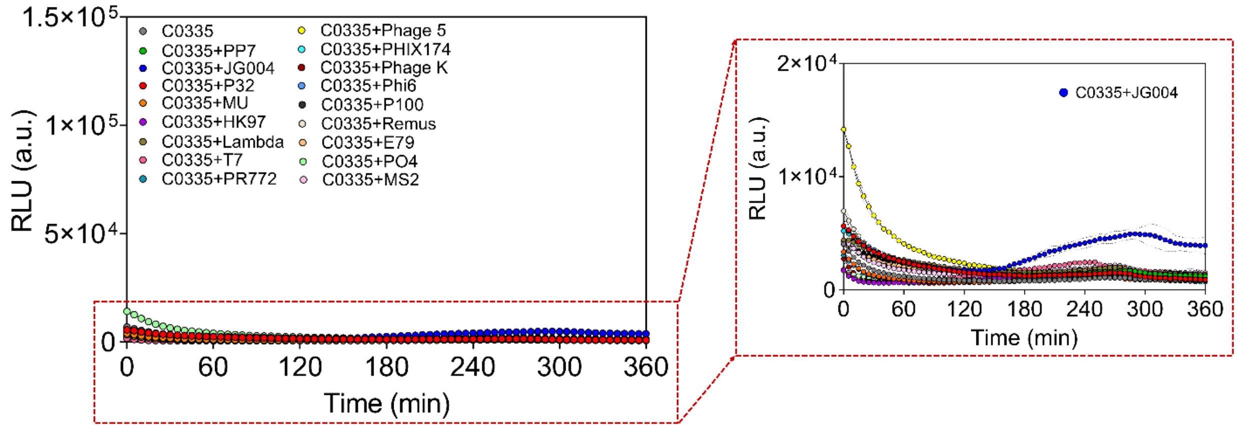


**Supplementary Figure 10.** Zoomed in image of the phage library screening, showing a delayed increase in bioluminescence signal for C0035 infected with JG004 appearing after 2 hours. The ATP assay was conducted in triplicates (n=3). The dashed lines show standard deviation from the mean. Source data are provided in Source Data file.

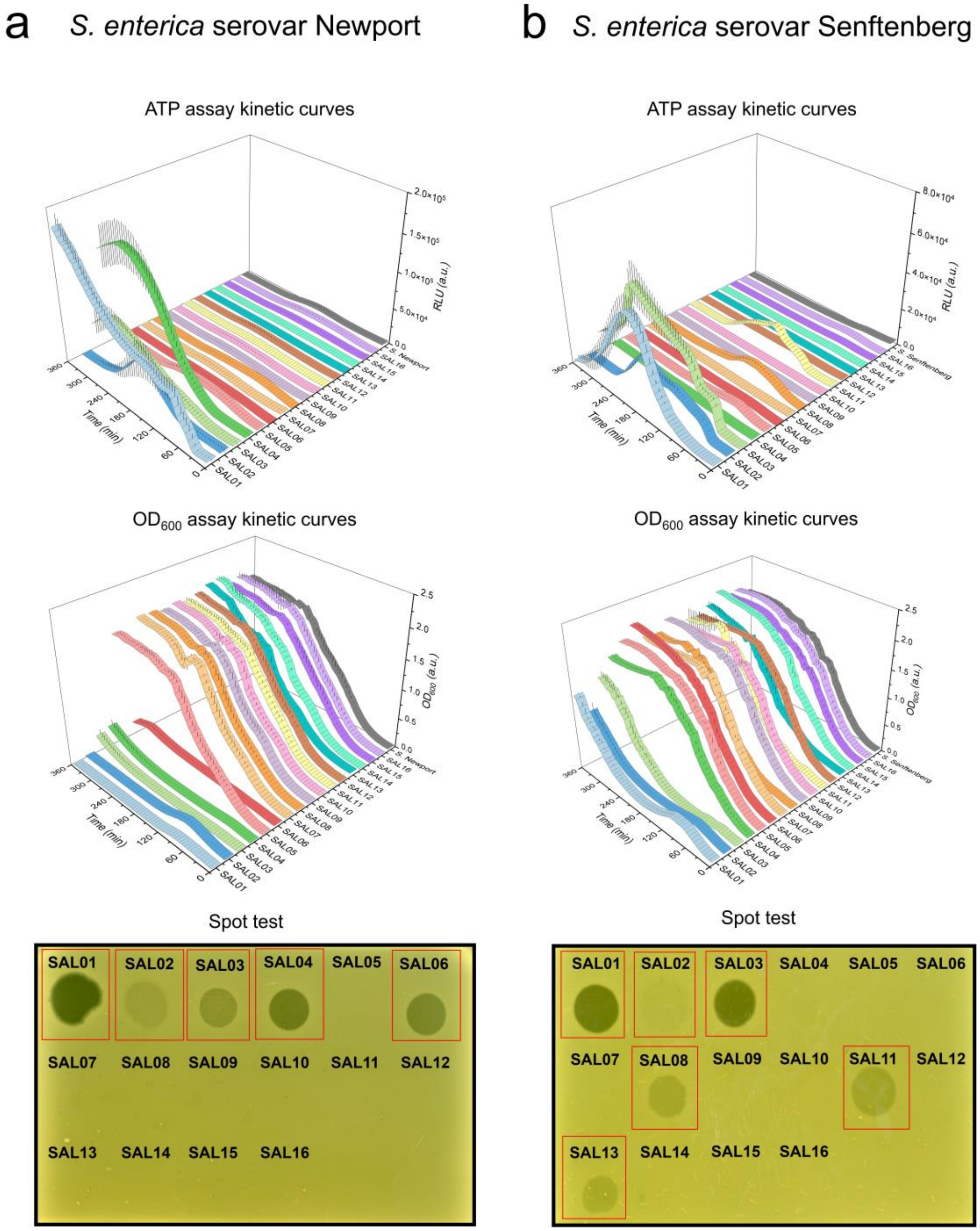


**Supplementary Figure 11. ATP and $OD_{600}$ kinetic curves and spot test of two *Salmonella* strains against 16 *Salmonella* phages. a** *Salmonella enterica* serovar Newport (*S. enterica* serovar Newport). **b** *Salmonella enterica* serovar Senftenberg (*S. enterica* serovar Senftenberg). ATP one-pot assay was conducted in the presence of sugar polymers. The ATP and $OD_{600}$ assays were conducted in triplicates (n=3), spot tests were repeated independently twice. The error bars show standard deviation from the mean. Source data are provided in Source Data file.

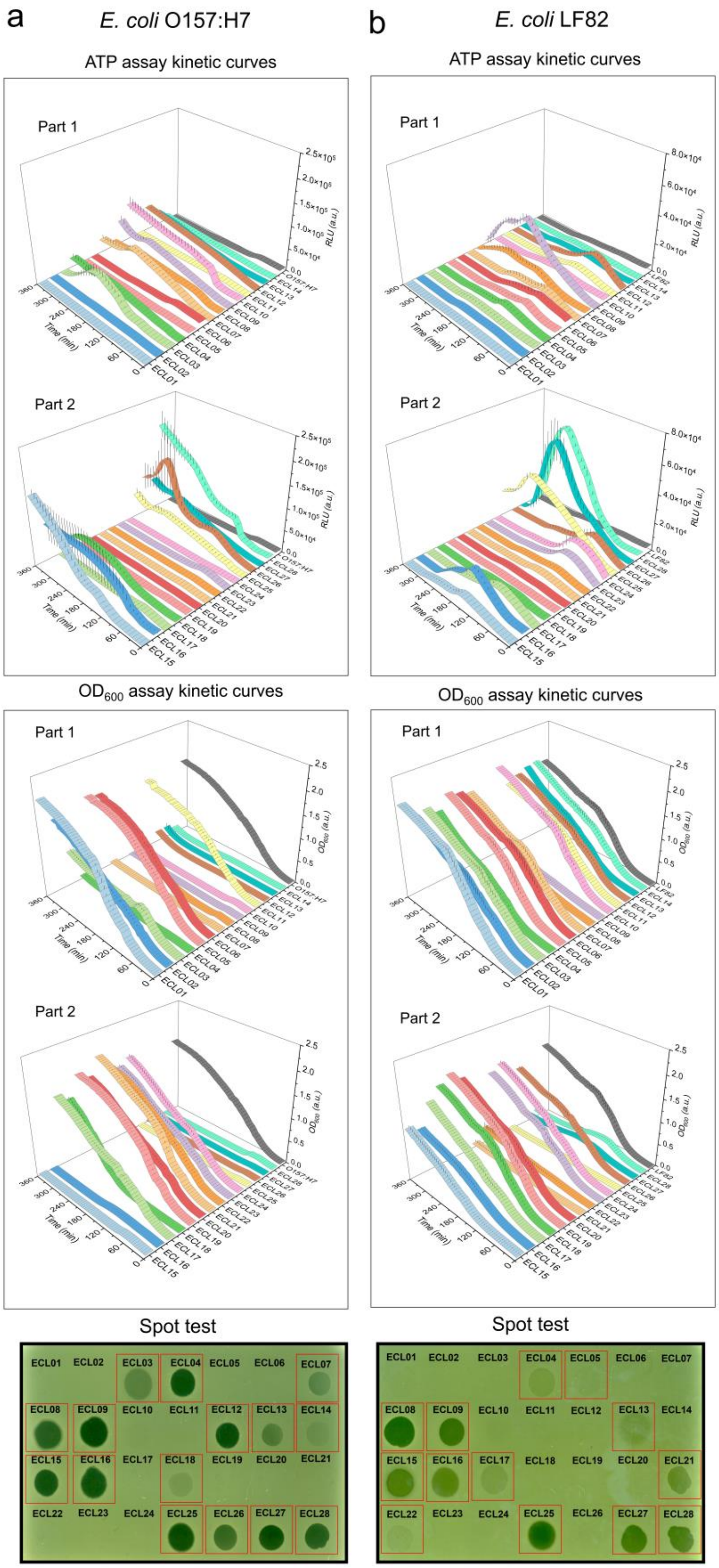
a
E. coli O157:H7
ATP assay kinetic curves
Part 1
Part 2
OD600 assay kinetic curves
Part 1
Part 2
Spot test
b
E. coli LF82
ATP assay kinetic curves
Part 1
Part 2
OD600 assay kinetic curves
Part 1
Part 2
Spot test

**Supplementary Figure 12. ATP and $OD_{600}$ kinetic curves and spot test of two *E. coli* strains against 28 *E. coli* phages. a** *E. coli* O157:H7 strain (human fecal isolate). **b** LF82 (Crohn's disease isolate[1]). ATP one-pot assay was conducted in the presence of sugar polymers. The ATP and $OD_{600}$ assays were conducted in triplicates (n=3). The error bars show standard deviation from the mean. Source data are provided in Source Data file.

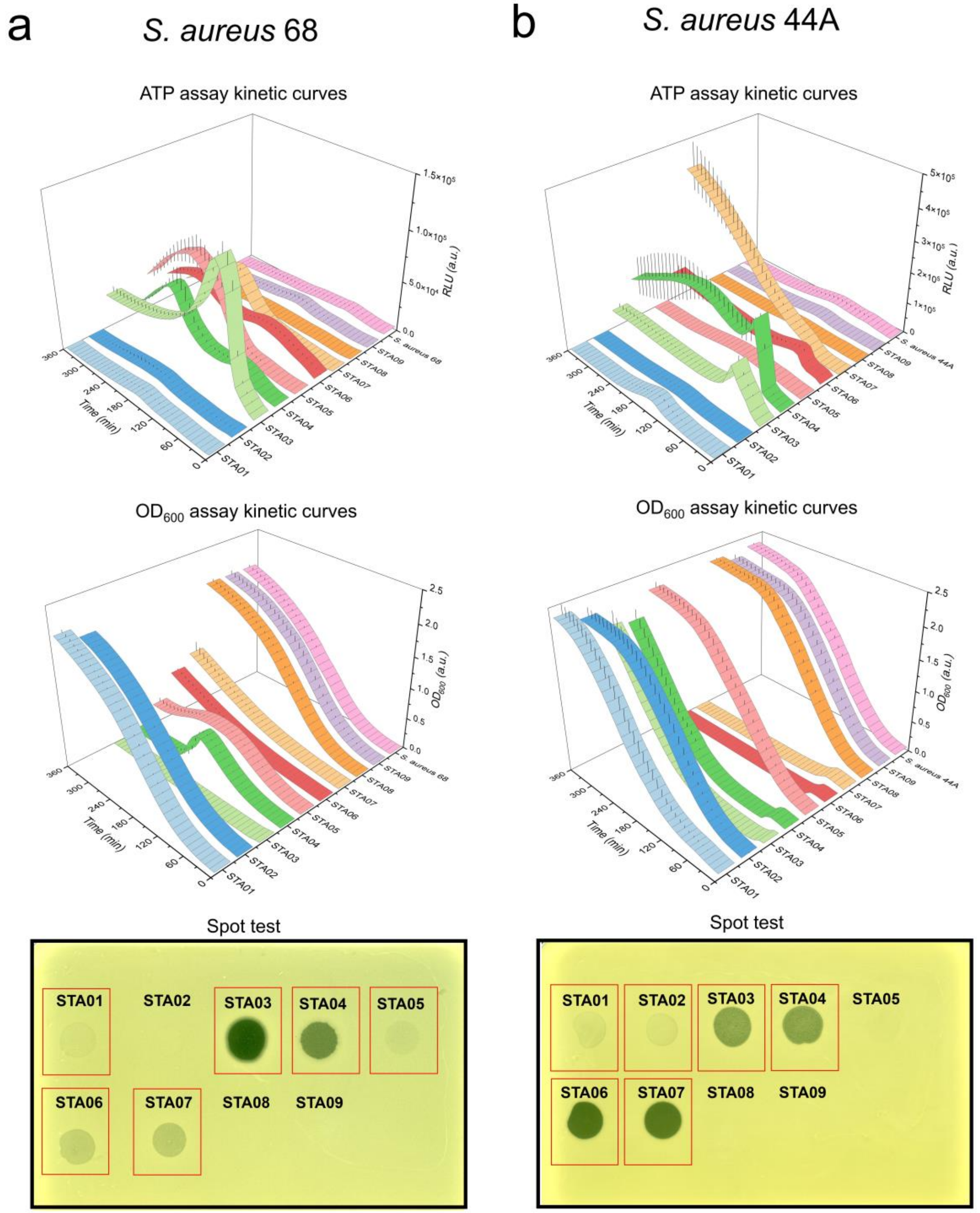


**Supplementary Figure 13. ATP and $OD_{600}$ kinetic curves and spot test of two *S. aureus* strains against nine *S. aureus* phages. a** HER 1049 *S. aureus* 68 strain. **b** HER 1101 *S. aureus* 44A. ATP one-pot assay was conducted in the presence of sugar polymers. The ATP and $OD_{600}$ assays were

conducted in triplicates (n=3). The error bars show standard deviation from the mean. Source data are provided in Source Data file.

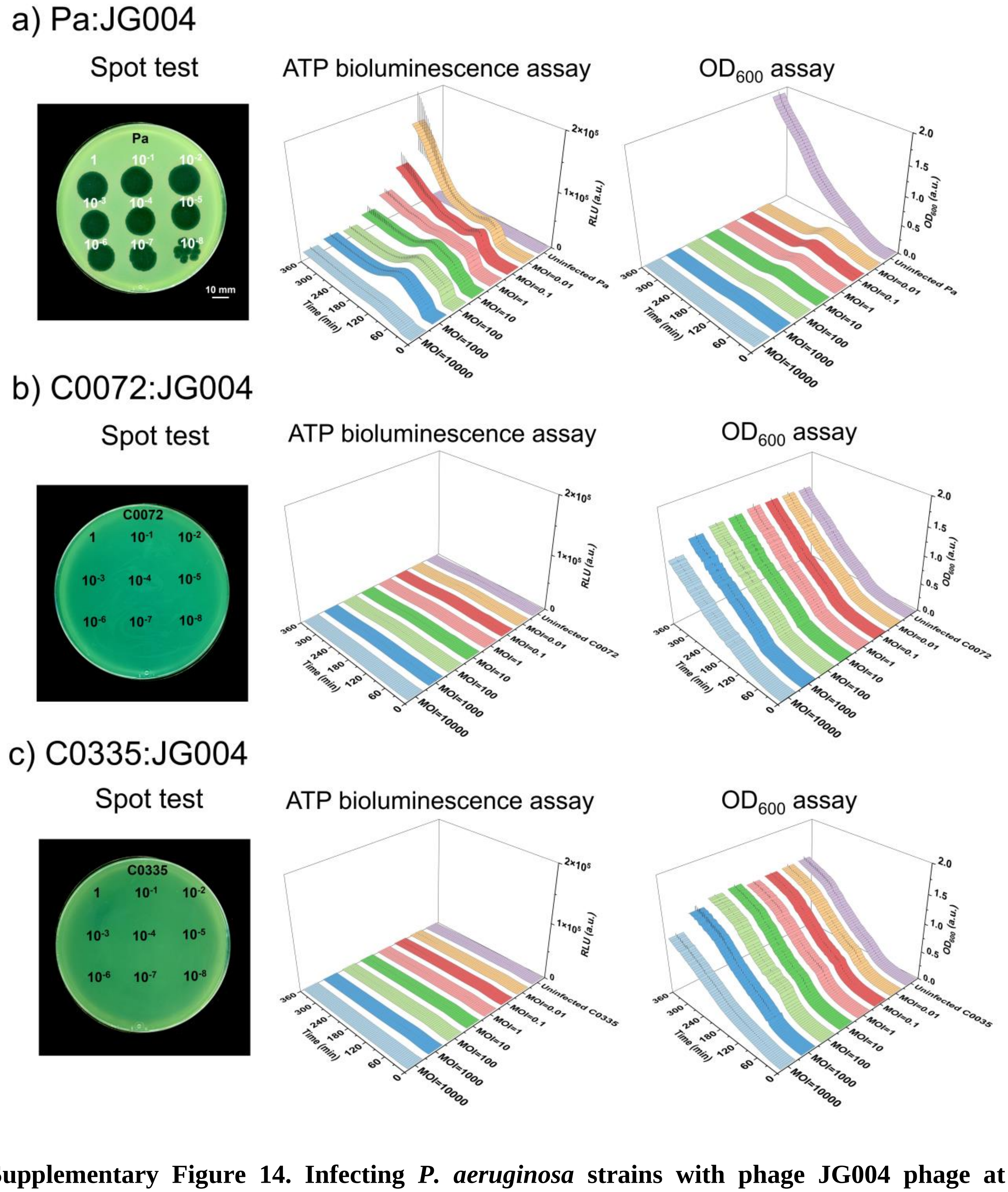


**Supplementary Figure 14. Infecting *P. aeruginosa* strains with phage JG004 phage at different MOIs.** Original phage JG004 titer was ~$10^{11}$ PFU/mL, which was diluted and spotted on the bacteria lawns. Dilution factors are shown on spot test plates ranging from 1 to

$10^{-8}$($\sim 5 \times 10^{11}$ PFU/mL to ~$5 \times 10^{3}$ PFU/mL). For the ATP and $OD_{600}$ assays, bacterial subcultures were infected with phage JG004 at MOIs of 10,000, 1000, 100, 10, 1, 0.1, 0.01. **Panel a)** Pa:JG004, **b)** C0072:JG004, **c)** C0335:JG004. The ATP and $OD_{600}$ assays were conducted in triplicates (n=3). The error bars show standard deviation from the mean. Source data are provided in Source Data file.

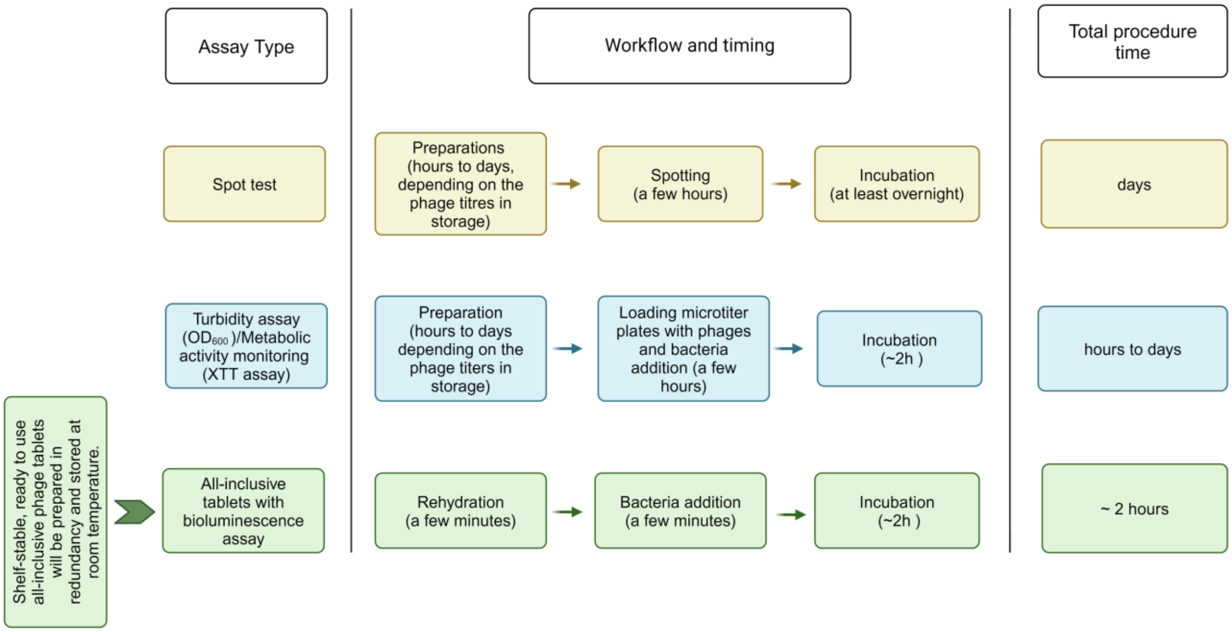


**Supplementary Figure 15.** Flowchart illustrating the overall time needed for susceptibility screening with various methodologies, namely spot test, kill curve, and the new proposed method, for a library of ~100 phages and one person dedicated to the task.

**Supplementary Table 1.** List of bacterial species and strains

| Bacteria | | Identifier | Source | Note |
|---|---|---|---|---|
| *P. aeruginosa* | PAO1 | DSM 22644 | DSMZ | NA |
| | C0072[2] | NA | Urine∗ | Antibiotic resistance profile: ampicillin, amoxicillin clavulanic acid, cefazolin, cefalotin, cefixime, nitrofuratoin, tetracycline, trimethoprim sulfamethoxazole, cefoxitin, ceftriaxone |
| | C0335[2] | NA | Arm∗ | ampicillin, amoxicillin clavulanic acid, cefazolin, cefalotin, cefixime, nitrofuratoin, tetracycline, trimethoprim sulfamethoxazole, cefoxitin, ceftriaxone |
| *Salmonella* | S. Newport C487 | HER1019 | Sewage ∗∗ | NA |
| | S. Senftenberg S-219/89 | HER1397 | Human blood ∗∗ | NA |
| *E. coli* | O157:H7 B1190-1 | HER1262 | Feces∗∗ | NA |
| | LF82 | NA | Ileal Mucosa of Crohn's disease patient[1], provided by Dr. Brian Coombes | NA |
| *S. aureus* | *S. aureus* 68 | HER 1049 | NA∗∗ | NA |
| | *S. aureus* 44A | HER 1101 | NA∗∗ | NA |

∗ Clinical isolates from the in-house library at the Michael DeGroote Institute of Infectious Disease Research.

∗∗ From the Felix D'Herelle Reference Center for Bacterial Viruses

**Supplementary Table 2.** List of *S. enterica* phages

| Phages | Codes |
|---|---|
| HER535 S16 | SAL01 |
| HER 40 01 | SAL02 |
| STL4 | SAL03 |
| HER20 16 | SAL04 |
| HER95 X | SAL05 |
| HER19 7-11 | SAL06 |
| HER428 1 Heidelberg | SAL07 |
| HER415 SasL6 | SAL08 |
| HER39 Vill | SAL09 |
| HER431 4 Heidelberg | SAL10 |
| HER397 SasL4 | SAL11 |
| ER429 2 Heidelberg | SAL12 |
| HER437 10 Heidelberg | SAL13 |
| HER340 Sab2 | SAL14 |
| HER161 P22 | SAL15 |
| HER45 Jersey | SAL16 |

**Supplementary Table 3.** List of *E. coli* phages

| Phage name | Codes | Phage name | Codes |
|---|---|---|---|
| HER139 HK243 | ECL01 | HER256 2 | ECL15 |
| HER144 Lambda | ECL02 | HER255 1 | ECL16 |
| HER263 9 | ECL03 | HER586 BRET | ECL17 |
| HER261 7 | ECL04 | HER468 T1uV | ECL18 |
| HER26 T3 | ECL05 | HER315 Ω8 | ECL19 |
| HER24 T1 | ECL06 | HER337 O103 | ECL20 |
| HER158 RB69 | ECL07 | HER42 pt1 | ECL21 |
| HER77 N4 | ECL08 | HER27 T4 | ECL22 |
| HER268 14 | ECL09 | HER382 HK97 | ECL23 |
| HER270 16 | ECL10 | HER364 SS4 | ECL24 |
| HER536 Alpha3 | ECL11 | HER258 4 | ECL25 |
| HER267 13 | ECL12 | HER257 3 | ECL26 |
| HER266 12 | ECL13 | HER259 | ECL27 |
| HER265 11 | ECL14 | HER262 | ECL28 |

**Supplementary Table 4.** List of *S. aureus* phages

| Phage name | Codes |
|---|---|
| HER225 3A | STA01 |
| HER226 77 | STA02 |
| HER49 P68 | STA03 |
| HER101 44AHJD | STA04 |
| HER528 Remus | STA05 |
| HER474 K | STA06 |
| HER475 812 | STA07 |
| HER239 187 | STA08 |
| HER238 71 | STA09 |

**Supplementary Table 5.** Comparative rating of the ATP detection method with culture techniques for *Salmonella* phages

| *Salmonella enterica* Serovar Newport | | | |
|---|---|---|---|
| Targets identified | ATP detection | OD kill curve | Spot test |
| SAL01 | +++ | +++ | +++ |
| SAL02 | ++ | +++ | + |
| SAL03 | ++ | +++ | + |
| SAL04 | +++ | +++ | ++ |
| SAL06 | No | +++ | ++ |
| *Salmonella enterica* Serovar Senftenberg | | | |
| SAL01 | +++ | +++ | +++ |
| SAL02 | ++ | +++ | - |
| SAL03 | +++ | +++ | +++ |
| SAL08 | + | + | + |
| SAL11 | + | + | + |
| SAL13 | - | + | + ∗ |

+++: strong signal (ATP assay: the highest and continuous RLU signal detected upon strain infection within the phage library, $OD_{600}$: the lowest turbidity detected upon strain infection within the phage library in comparison with negative control (uninfected strain), Spot test: clear plaque on the strain's bacterial lawn)
++: medium signal (ATP assay: a medium RLU signal detected upon strain infection in comparison with the highest RLU value within the phage library, $OD_{600}$: the medium reduction in turbidity detected upon strain infection in comparison with negative control, Spot test: turbid plaque on the strain's bacterial lawn)
+: weak signal (ATP assay: a weak RLU signal value detected upon strain infection in comparison with the highest and medium RLU value within the phage library, $OD_{600}$: a weak reduction in turbidity detected upon strain infection in comparison with negative control, Spot test: faint plaque on the strain bacterial lawn
∗: very faint footprint

**Supplementary Table 6.** Comparative rating of the ATP detection method with culture techniques for *E. coli* phages

| *E. coli O157: H7* | | | |
|---|---|---|---|
| Targets identified | ATP detection | OD kill curve | Spot test |
| ECL03 | + | + | + |
| ECL04 | + | +++ | +++ |
| ECL07 | + | +++ | + |
| ECL08 | + | +++ | +++ |
| ECL09 | + | +++ | +++ |
| ECL10 | ++ | +++ | NA * |
| ECL12 | + | +++ | +++ |
| ECL13 | + | +++ | + |
| ECL14 | + | +++ | + |
| ECL15 | +++ | +++ | +++ |
| ECL16 | +++ | +++ | +++ |
| ECL18 | ++ | ++ | + |
| ECL25 | + | +++ | +++ |
| ECL26 | +++ | +++ | ++ |
| ECL27 | + | +++ | +++ |
| ECL28 | +++ | +++ | +++ |
| *E. coli LF82* | | | |
| ECL04 | + | + | + |
| ECL07 | + | No | No |
| ECL08 | + | + | +++ |
| ECL09 | ++ | ++ | +++ |
| ECL12 | + | No | No |
| ECL13 | No | No | + ** |
| ECL15 | + | ++ | ++ |
| ECL16 | + | ++ | ++ |
| ECL17 | + | + | + |
| ECL21 | No | +++ | + *** |
| ECL22 | No | +++ | + *** |
| ECL23 | + | No | No |
| ECL24 | + | No | No |
| ECL25 | +++ | +++ | +++ |
| ECL26 | + | No | No |
| ECL27 | +++ | +++ | +++ |
| ECL28 | +++ | +++ | +++ |

+++: strong signal (ATP assay: the highest and continuous RLU signal detected upon strain infection within the phage library, $OD_{600}$: the lowest turbidity detected upon strain infection within the phage library in comparison with negative control (uninfected strain), Spot test: clear plaque on the strain's bacterial lawn)
++: medium signal (ATP assay: a medium RLU signal detected upon strain infection in comparison with the highest RLU value within the phage library, $OD_{600}$: the medium reduction in

turbidity detected upon strain infection in comparison with negative control, Spot test: turbid plaque on the strain's bacterial lawn)
+: weak signal (ATP assay: a weak RLU signal value detected upon strain infection in comparison with the highest and medium RLU value within the phage library, $OD_{600}$: a weak reduction in turbidity detected upon strain infection in comparison with negative control, Spot test: faint plaque on the strain bacterial lawn
*: The kill curve and ATP detection methods exhibit relatively strong signals, leading to the conclusion that the spot test in this case may be biased.

**: very faint footprint
***: no plaques detected upon serial dilution.

**Supplementary Table 7.** Comparative rating of the ATP detection method with culture techniques for *S. aureus* phages

| *S. aureus 68* | | | |
|---|---|---|---|
| Targets identified | ATP detection | OD kill curve | Spot test |
| STA01 | No | No | +* |
| STA03 | +++ | +++ | +++ |
| STA04 | ++ | ++ | ++ |
| STA05 | ++ | ++ | +* |
| STA06 | ++ | ++ | +* |
| STA07 | ++ | ++ | +* |
| *S. aureus 44A* | | | |
| STA01 | No | + | +* |
| STA02 | No | + | +* |
| STA03 | ++ | ++ | ++ |
| STA04 | +++ | ++ | ++ |
| STA06 | ++ | +++ | ++ |
| STA07 | +++ | +++ | ++ |

+++: strong signal (ATP assay: the highest and continuous RLU signal detected upon strain infection within the phage library, $OD_{600}$: the lowest turbidity detected upon strain infection within the phage library in comparison with negative control (uninfected strain), Spot test: clear plaque on the strain's bacterial lawn)
++: medium signal (ATP assay: a medium RLU signal detected upon strain infection in comparison with the highest RLU value within the phage library, $OD_{600}$: the medium reduction in turbidity detected upon strain infection in comparison with negative control, Spot test: turbid plaque on the strain's bacterial lawn)
+: weak signal (ATP assay: a weak RLU signal value detected upon strain infection in comparison with the highest and medium RLU value within the phage library, $OD_{600}$: a weak reduction in turbidity detected upon strain infection in comparison with negative control, Spot test: faint plaque on the strain bacterial lawn
*: very faint footprint

## References


1. Darfeuille-Michaud, A. *et al.* Presence of adherent *Escherichia coli* strains in ileal mucosa of patients with Crohn's disease. *Gastroenterology* **115**, 1405–1413 (1998).
2. Alcock et al. 2023. CARD 2023: Expanded Curation, Support for Machine Learning, and Resistome Prediction at the Comprehensive Antibiotic Resistance Database. Nucleic Acids Research, 51, D690-D699.
