# Supplementary material for: High throughput platform technology for rapid target identification in personalized phage therapy
Source: Nat Commun. 2024 Jul 11;15:5626. doi: 10.1038/s41467-024-49710-2 (PMC11239838; doi:10.1038/s41467-024-49710-2)
Supplement: Supplementary file 3 — Reporting Summary [file 41467_2024_49710_MOESM3_ESM.pdf]

nature portfolio

Corresponding author(s): Zeinab hosseinidoust, Tohid F. Didar

Last updated by author(s): Jun 7, 2024

Reporting Summary

Nature Portfolio wishes to improve the reproducibility of the work that we publish. This form provides structure for consistency and transparency in reporting. For further information on Nature Portfolio policies, see our Editorial Policies and the Editorial Policy Checklist.

Statistics

For all statistical analyses, confirm that the following items are present in the figure legend, table legend, main text, or Methods section.

n/a	Confirmed	
☐	☒	The exact sample size (*n*) for each experimental group/condition, given as a discrete number and unit of measurement
☐	☒	A statement on whether measurements were taken from distinct samples or whether the same sample was measured repeatedly
☐	☒	The statistical test(s) used AND whether they are one- or two-sided<br>*Only common tests should be described solely by name; describe more complex techniques in the Methods section.*
☐	☒	A description of all covariates tested
☒	☐	A description of any assumptions or corrections, such as tests of normality and adjustment for multiple comparisons
☐	☒	A full description of the statistical parameters including central tendency (e.g. means) or other basic estimates (e.g. regression coefficient) AND variation (e.g. standard deviation) or associated estimates of uncertainty (e.g. confidence intervals)
☐	☒	For null hypothesis testing, the test statistic (e.g. *F*, *t*, *r*) with confidence intervals, effect sizes, degrees of freedom and *P* value noted<br>*Give P values as exact values whenever suitable.*
☒	☐	For Bayesian analysis, information on the choice of priors and Markov chain Monte Carlo settings
☒	☐	For hierarchical and complex designs, identification of the appropriate level for tests and full reporting of outcomes
☒	☐	Estimates of effect sizes (e.g. Cohen's *d*, Pearson's *r*), indicating how they were calculated

Our web collection on statistics for biologists contains articles on many of the points above.

Software and code

Policy information about availability of computer code

Data collection	Data for this paper was collected from Transmission Electron Microscopy (Talos L120C), plate reader (Synergy Neo2 BioTek). No external custom code or algorithm was used.
Data analysis	Graphs were mainly created using GraphPad Prism (version 9.5.1 for Windows, GraphPad Software, San Diego, California USA, www.graphpad.com), except for figure 5, which was created in a 3D format using Biorender.com (the snapshot of each time point were based on data presented in the SI. 3D graphs in the supplementary information in SI Fig. 8, 11, 12, 13, and 14 were graphed using Origin Pro software (Version 2023. OriginLab Corporation, Northampton, MA, USA). No external custom code or algorithm was used.

For manuscripts utilizing custom algorithms or software that are central to the research but not yet described in published literature, software must be made available to editors and reviewers. We strongly encourage code deposition in a community repository (e.g. GitHub). See the Nature Portfolio guidelines for submitting code & software for further information.

Data

Policy information about availability of data

All manuscripts must include a data availability statement. This statement should provide the following information, where applicable:

- Accession codes, unique identifiers, or web links for publicly available datasets
- A description of any restrictions on data availability
- For clinical datasets or third party data, please ensure that the statement adheres to our policy

A source data file (a single excel file with data for each figure in a spreadsheet)will be provided with this paper.

Research involving human participants, their data, or biological material

Policy information about studies with human participants or human data. See also policy information about sex, gender (identity/presentation), and sexual orientation and race, ethnicity and racism.

Reporting on sex and gender	NA
Reporting on race, ethnicity, or other socially relevant groupings	NA
Population characteristics	NA
Recruitment	NA
Ethics oversight	NA

Note that full information on the approval of the study protocol must also be provided in the manuscript.

Field-specific reporting

Please select the one below that is the best fit for your research. If you are not sure, read the appropriate sections before making your selection.

☒ Life sciences ☐ Behavioural & social sciences ☐ Ecological, evolutionary & environmental sciences

For a reference copy of the document with all sections, see nature.com/documents/nr-reporting-summary-flat.pdf

Life sciences study design

All studies must disclose on these points even when the disclosure is negative.

Sample size	Sample sizes were chosen to be the minimum for meaningful statistical analyses (i.e., n=3 or higher) which provided robust differences or similarities between conditions and sample groups. The sample size is n>=3 for each assay including ATP assay, OD assay, and XTT assay and for CFU and PFU counts.<br>No statistical method was used to predetermine sample size
Data exclusions	No data were excluded from the analyses. The only thing to consider is that when analyzing one pot liquid ATP assay results, in some cases with non-target phages, the bioluminescence signal was so low that after background subtraction the value was negative. This negative number has not physical significance and is merely noise, therefore, it does not affect the result of screening process, does not compromise the final results, or change any trends.
Replication	For all experiments, the initial bacterial cultures and phage concentration, the CFU and PFU counts were calculated using at least 3 technical replicates, each plated at least 3 times.<br>All attempts at replication were successful.<br>Figure 1d- The XTT assay were performed in 3 independent experiments (3 biological replicates), each with 6 technical replicates<br>Figure 1c- SEM imaging of the phages were repeated independently at least twice.<br>Figure 2b to g - The End point ATP measurements were conducted in 3 independent experiments, each with 3 technical replicates<br>Figure 3b - The kinetic one-pot ATP assays were conducted with 3 biological replicates each with 3 technical replicates. Figure 3d and Figure 3c were conducted in 3 independent experiments (3 biological replicates) each with 3 technical replicates.<br>Figure 4b, c, d, e - The one-pot ATP assays in the presence of pullulan and trehalose were conducted in 3 independent experiments (3 biological replicates) and 3 technical replicates<br>Figure 4f and g - The stability of the phage and ATP assay reagents without adding any sugars were tested by 3 biological replicates each with 3 technical replicates.<br>Figure 5b, c, d, e. The final screening of the clinical strains against the in house phage library, 3 technical replicates per phage was used.<br>Supplementary Fig. 1- The optical density assays for each phage was performed in 3 independent experiments with 3 biological replicates each with 6 technical replicates<br>Supplementary Fig. 3 - The background ATP present at different steps of purification and after phage dilutions was measured with 3 technical replicates

Supplementary Fig. 4,5. Effect of sugar polymers on stability of the ATP reagent solution at 37°C was evaluated with 3 technical replicates
Supplementary Fig. 6 - The stability of phages in sugar polymer matrix and no sugar added was tested using 3 technical replicates, each plated 3 times to obtain the phage titer
Supplementary Fig. 7. Phage and ATP reagents stability assay after 4 weeks was assessed with 6 technical replicates
Supplementary Fig. 8a,b - The optical density and one-pot ATP bio luminescence assays of the infected and uninfected P. aeruginosa clinical strains were tested using one biological replicate, with 3-6 technical replicates
Supplementary Fig. 9 - The XTT assays of the infected and uninfected clinical strains were conducted using one biological replicate with 3-6 technical replicates
Supplementary Fig. 10. is a zoomed in image of Supplementary Fig.8b, conducted with three technical replicates.
Supplementary Fig. 11. ATP and OD600 kinetic curves and spot test of two Salmonella strains against 16 Salmonella phages were conducted with three technical replicates.
Supplementary Fig. 12. ATP and OD600 kinetic curves and spot test of two E. coli strains against 28 E. coli phages were conducted with three technical replicates.
Supplementary Fig. 13. ATP and OD600 kinetic curves and spot test of two S. aureus strains against 9 S. aureus phages conducted with three technical replicates
Supplementary Fig. 14. ATP and OD600 kinetic curves (condcuted with three technical replicates and spot test of four P. aeruginosa strains including Pa (PAO1), C0072 and C0335 infected with JG004 phage at different MOIs.

Randomization

Allocation was not random, we paired bacteria along with libray phages that where classified to target bacteria in the same species (figure 5) except for 5b, and c where the bacteria was allocated to a collection of phages some of which where known to infect the same species and some that where known to infect other species, as a means to challenge the system and look for false positives.
in figures 1-4, bacteria was assigned to phage known to infect it through spot tests.

Blinding

Since we were exploring the effectiveness of the ATP assay in detecting phage-mediated bacterial cell lysis, the experiments were conducted with a group of Pseudomonas phages and their host, Pseudomonas aeruginosa PAO1 (Pa). Some of the OD and XTT assays were conducted by Arwa Hilal, without being informed of the results from other assays. We also received a collection of phages from the Felix d'Herelle Reference Center for Bacterial Viruses at University Laval (https://www.phage.ulaval.ca/en/home/), prepared by Denise Tremblay.

Reporting for specific materials, systems and methods

We require information from authors about some types of materials, experimental systems and methods used in many studies. Here, indicate whether each material, system or method listed is relevant to your study. If you are not sure if a list item applies to your research, read the appropriate section before selecting a response.

Materials & experimental systems

n/a	Involved in the study
☒	☐ Antibodies
☒	☐ Eukaryotic cell lines
☒	☐ Palaeontology and archaeology
☒	☐ Animals and other organisms
☒	☐ Clinical data
☒	☐ Dual use research of concern
☒	☐ Plants

Methods

n/a	Involved in the study
☒	☐ ChIP-seq
☒	☐ Flow cytometry
☒	☐ MRI-based neuroimaging
